# Lipid Production From Waste Materials in Seawater-Based Medium by the Yeast *Yarrowia lipolytica*

**DOI:** 10.3389/fmicb.2019.00547

**Published:** 2019-03-18

**Authors:** Adam Dobrowolski, Katarzyna Drzymała, Dorota A. Rzechonek, Paweł Mituła, Aleksandra M. Mirończuk

**Affiliations:** ^1^Department of Biotechnology and Food Microbiology, Wrocław University of Environmental and Life Sciences, Wrocław, Poland; ^2^Institute of Environmental Engineering, Wrocław University of Environmental and Life Sciences, Wrocław, Poland

**Keywords:** seawater, lipid production, crude glycerol, *Yarrowia lipolytica*, single cell oil

## Abstract

The global limitation of fossil fuels impels scientists to search for new energy sources. A good alternative is biodiesel produced from crop plants. However, its production requires huge quantities of farmland, fertilizers and fresh water, which is in conflict with the human demand for water for consumption and land for food production. Thus, production of single cell oil (SCO) by oleaginous microorganisms remains the best solution for the coming years. Whereas most microorganisms require fresh water for proper cell metabolism, in this study we demonstrate that the unconventional yeast *Yarrowia lipolytica* is able to produce huge quantities of fatty acid in seawater-based medium. Here we shown that *Y. lipolytica* is able to produce fatty acids in medium based on seawater and crude glycerol as the main carbon source, which allows for low-cost production of SCO, is beneficial for industrial application and is ecologically friendly.

## Introduction

The yeast *Yarrowia lipolytica* is a well-studied microorganism used as a model for lipid synthesis in eukaryotic cells (Lazar et al., 2018). *Y. lipolytica* naturally occurs in food such as cheese and meat, but also has been found in soil and marine environments (Groenewald et al., 2014). This yeast is also known for its ability to produce organic acids (Rymowicz et al., 2010; Otto et al., 2012; Kamzolova et al., 2018), polyols (Rakicka et al., 2016; Janek et al., 2017; Rzechonek et al., 2017a) and biomass (Juszczyk et al., 2013) from untypical carbon sources such as crude glycerol, n-alkanes or agro-industrial residues (Papanikolaou et al., 2002, 2003; Dobrowolski et al., 2016). This feature allows for biosynthesis of value-added products from low-cost substrates. All these features cause that *Y. lipolytica* is an attractive host for industrial applications.

One of the important issues of the coming years is the decreasing amount of fossil fuels and humanity’s dependence on the fuels in everyday life. To solve this issue, for many years production of biodiesel from crop plants (sunflowers, canola) has been applied on a large scale. This in turn raises other issues. Direct use of vegetable oils is unsatisfactory and impractical for direct-injection and indirect-type diesel engines. Thus biodiesel is derived from triglycerides by transesterification with methanol, generating the side-product glycerol (Fukuda et al., 2001). However, production of biodiesel from crops plants requires huge areas of farmlands and fertilizers and enormous quantities of fresh water, and the yield is dependent on the environmental conditions. Moreover, there is a competition between food and biodiesel production, since most crop plants are intended for the food industry. Single cell oil (SCO) is a promising alternative as a biodiesel for replacing fossil fuels in future. In contrast to biodiesel based on crops plants, microbiological biofuel production is independent from the weather and allows control of lipid type and content via overexpression of key enzymes involved in certain lipid biosynthesis (Blazeck et al., 2014). Overexpression of the *DGA1* (YALI0E32769g) gene encoding an acylCoA diacylglycerol acyltransferase results in increased lipid production by *Y. lipolytica* (Athenstaedt, 2011; Qiao et al., 2015).

Despite the advantages, production of SCO is still not applied on the industrial scale. First, the production process has to be economically profitable, otherwise the interest of the refinery industry will be low. Therefore, the production medium has to be based on low-cost carbon sources, e.g., wastes from various industrial branches (agricultural, cosmetic or biodiesel production). The next issue that has to be solved is a requirement for fresh water in the industry. Despite the fact that the majority of the Earth’s surface is covered in water (about 70%), most of this is held by oceans and seas and due to the salinity is unavailable for humans and agriculture. On average, seawater in the world’s oceans has a salinity of about 3.5% (Wright and Colling, 1995). The amount of fresh water available for humans is very limited. Only about 0.01% of the world’s total water supply is available for human use on a regular basis. Moreover, globally about 70% of fresh water is used for agricultural purposes (Data World Bank, 2017). In Europe, industry is one of the main water users, accounting for about 40% of total water abstractions (Eurostat, 2017). Climate changes cause increasing fresh water issues, and the availability for human use is slowly decreasing. All this means that replacement of fresh water by seawater for industrial purposes will be an important issue in the coming years.

Therefore, to meet above expectations concerning industrial production of biofuels from microbial lipids we employed yeast *Y. lipolytica* to synthetize desired product in medium with seawater instead of fresh water and crude glycerol as a sole carbon source. The usage of seawater and glycerol causes a high osmotic pressure, which can be disturbing factor for microbial growth and lipids production. The aim of this study was to investigate the possibility of use the seawater to prepare lipid production medium for yeast *Y. lipolytica*. During the study we use a wild-type and modified strain AJD pADDGA1, overexpressing the DGA1 gene to increase the yield of lipid production. First, we tested the ability of both strains the wild-type and the engineered strain to grow on seawater-based medium. Next, we used crude glycerol as a sole carbon source, to investigated if the engineered strain can produce high lipid titer in the medium with elevated osmotic pressure. Next, we performed a bioreactor study to check if enlarging the production scale keep the lipid titer at a high level. This is the first study focusing on the use of seawater for lipid production by *Y. lipolytica*.

## Materials and Methods

### Yeast Strains

Strains used in this study were *Y. lipolytica* A101 (Wojtatowicz and Rymowicz, 1991), AJD (Mirończuk et al., 2015), and AJD pAD-DGA1. All strains belong to the Department of Biotechnology and Food Microbiology at Wrocław University of Environmental and Life Sciences, Poland.

### Media and Culture Conditions

*Escherichia coli* strains were cultivated in LB (Luria broth, BTL, Poland) medium according to standard protocols (Sambrook and Russel, 2001). Rich Yeast Extract Peptone Glucose (YPD) medium was used for the yeast inoculum preparation and contained: yeast extract 10 g/L (Merck, Germany), peptone 10 g/L (Biocorp, Poland) and glucose 20 g/L (Merck, Germany). The production medium for the lipid production experiments consisted of : YNB 1.7 g/L (Yeast Nitrogen Base without amino acids and ammonium sulfate, Sigma, Germany), supplemented with glucose 50 g/L (Merck, Germany) or pure glycerol 50 g/L (POCH, Poland) and (NH_4_)_2_SO_4_ 0.92 g/L, C/N ratio 100, pH 3.0. Medium for lipid synthesis based on crude glycerol: crude glycerol 50 g/L, (NH_4_)_2_ SO_4_ 1.57 g/L, MgSO_4_ × 7H_2_O 1 g/L, YE 0.5 g/L, C/N ratio 60, pH 3.0. To obtain a seawater based medium the sea salts mixture (Sigma-Aldrich) was added to medium in concentration 36 g/L to mimic a seawater (SW).

### Analytical Methods

Samples (10 ml) from the cultures were centrifuged (10 min; 4°C; 5500× g), harvested by filtration on 0.45-μm pore membranes and washed twice with distilled water. The biomass was determined gravimetrically after drying at 105°C. The concentrations of glycerol and citric acid (CA) were determined with HPLC using a HyperRez Carbohydrate H^+^ Column (Thermo Scientific, Waltham, MA, United States) coupled to a UV (λ = 210 nm) (Dionex, Sunnyvale, United States) and a refractive index (RI) detector (Shodex, Ogimachi, Japan). The column was eluted with 25 mM of trifluoroacetic acid (TFA) at 65°C and a flow rate of 0.6 ml min^-1^.

### Growth of *Y. lipolytica* in Bioscreen C

The yeast strain *Y. lipolytica* A101 was grown in 100-well plates in 200 μL of fresh water based medium or seawater-based medium, supplemented with glucose (YPD) or glycerol (YPGly) 2% (w/v). First, the A101 strain was grown for 24 h in YPD medium. Next, the cells were washed and inoculated to an OD_600_ of 0.1 in each well. Quintuple experiments were performed at 28°C under constant agitation with Bioscreen C (Oy Growth Curves Ab Ltd., Finland). Growth was monitored measuring optical density at 420–560 nm every 30 min for 72 h.

### Conditions During Shake-Flask Experiments

The growth medium used for the inoculum was rich YPD medium. The production medium for the shake-flask experiment consisted of YNB medium (Yeast Nitrogen Base without amino acids and ammonium sulfate, Sigma, Germany) based on crude glycerol (described in section above). During shake-flask experiments the cultures were grown in 0.3 L flasks containing 0.03 L of medium on a rotary shaker (CERTOMAT IS, Sartorius Stedim Biotech) at 30 C at 200 rpm for 120 h.

### Conditions During Bioreactor Experiments

To prepare an inoculation culture for SCO production in a bioreactor, the cultures were grown in 0.3 L flasks (containing 0.1 L of YPD medium) on a shaker at 28°C for 72 h, 200 rpm. An inoculum of 0.2 L was introduced into the bioreactor containing crude glycerol medium: fresh water, 150 g/L crude glycerol (187.5 g/L of 80% crude glycerol), 4.61 g/L (NH_4_)_2_SO_4_ in C/N ratio 60, 1 g/L yeast extract, 1 g/L MgSO_4_ × 7H_2_O (FW-CG medium) or seawater, 150 g/L crude glycerol (187.5 g/L of 80% crude glycerol), 4.61 g/L (NH_4_)_2_SO_4_ in C/N ratio 60, 1 g/L yeast extract, 1 g/L MgSO_4_ × 7H_2_O and 36 g/L of sea salts (Sigma-Aldrich) (SW-CG medium). The cultivations were performed in a 5-l jar bioreactor (B 92 iostat B Plus, Sartorius, Germany) with a working volume of 2 l at 28°C. The aeration was fixed at 0.6 l min^-1^. The stirrer speed was adjusted to 800 rpm, and the dissolved oxygen concentration was maintained at 25 ± 5%. The pH was maintained automatically at 3.0 by addition of NaOH (20% w/v). During the batch cultures, evaporation is limited because the exhaust gasses pass into the exhaust condenser where moisture is removed and returned to the vessel. The cultures were performed in three replicates.

### Lipid Extraction and Fatty Acid Characterization

The fatty acids (FAs) from lyophilized biomass were derivatized to fatty acid methyl esters (FAMEs) using the method described before (Browse et al., 1986). Briefly, biomass (approximately 10–20 mg) was mixed with 2 ml of 2.5% sulfuric acid in methanol (containing 50 μg/ml of C17:0 as an internal standard) in glass tubes with a Teflon cap, vigorously mixed for 2 min and incubated at 80°C for 90 min to form FAMEs. FAMEs were extracted by adding 1 ml of hexane and 0.5 ml of water, mixed and spun down (centrifuged) for better phase separation. The organic phase containing FAMEs was transferred into glass vials for GC analysis. FAMEs were analyzed by gas chromatography on GC-2010 Plus apparatus (Shimadzu, Japan) with a flame ionization detector (FID) and autoinjector (AOC-20i). The separation of FAMEs was achieved using a 70% cyanopropyl polysilphenylene-siloxane column (TR-FAME, 30 m × 0.32 mm × 0.25 μm). The initial oven temperature was 130°C held for 1 min, which was then increased to 200°C at the rate of 5°C × min^-1^, then a rise to 250°C at a rate of 10°C × min^-1^ and held for 1 min. Temperatures for the injector and detector were 270 and 280°C, respectively. Helium was used as the carrier gas with constant flow of 1.52 mL × min^-1^. The volume of injection was 1 μL with a split rate of 1:5.

The identification of FAME was evaluated using Supelco 37 Component Fame Mix as a reference standard, and for quantification analysis heptanoic acid was used as an internal standard. The total lipid content in the dry cell weight was calculated as the sum of all fatty acids.

### Construction of Overexpression Strains

The gene coding *DGA1*(YALI0E32769g) was amplified from *Y. lipolytica* genomic DNA with primers DGA1_AscI_F: GCATGGCGCGCCATGACTATCGACTCACAATAC and DGA1_NheI_R: ATGCGCTAGCGCCTAACCCAGGCAGTTTTC, resulting in a 1588 bp PCR fragment. It was digested with the enzymes *AscI* and *NheI* and cloned into corresponding sites of the plasmid pAD (Mirończuk et al., 2017), carrying the UAS1B16-TEF promoter and XPR2 terminator. The obtained construct pAD-DGA1 was sequenced (Genomed, Poland). The obtained plasmid pAD-DGA1 was digested with *MssI* to create linear expression cassettes devoid of *E. coli* DNA and surrounded by *Y. lipolytica* rDNA for targeted integrations. It was used to transform *Y. lipolytica* AJD resulting in the strain AJD pAD-DGA1. The obtained strain AJD pAD-DGA1 was confirmed for correct integration through gDNA extraction and three distinct PCR confirmations.

## Results and Discussion

### Growth of *Y. lipolytica* in Seawater-Based Medium

**FIGURE 1:**
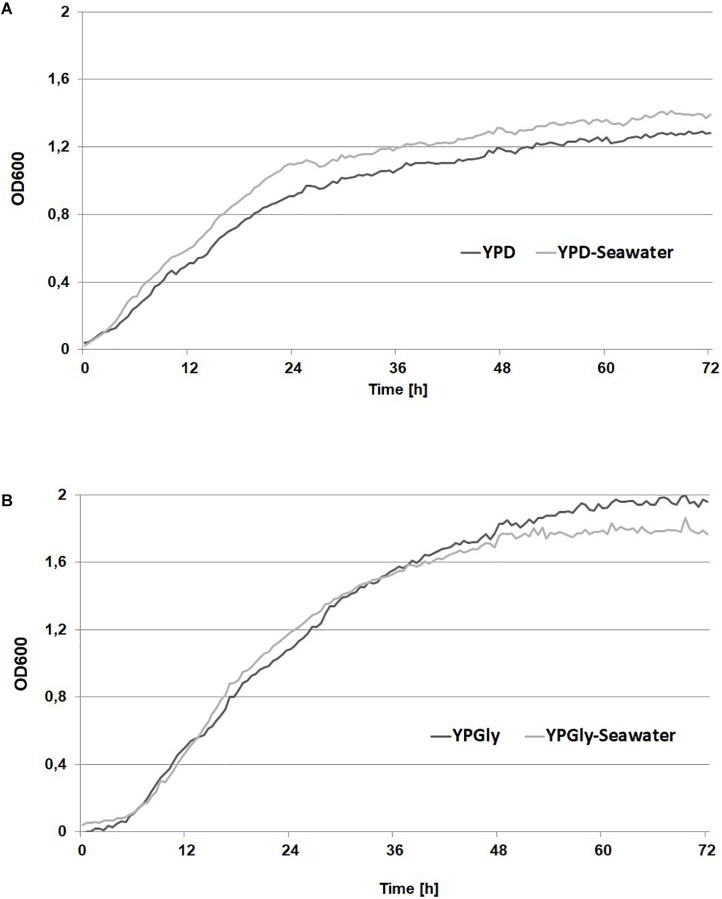
Growth curves of *Yarrowia lipolytica* A 101 in medium based on freshwater (dark gray) and seawater (light gray) based medium. Strains were grown on YPD medium **(A)** containing 2% glucose or YPGly medium **(B)** containing 2% glycerol as carbon source. The seawater based medium contain 36 g/L of sea salts mixture. Experiment was performed at 28°C under constant agitation using Bioscreen C.

Natural habitats of yeast *Y. lipolytica* are lipid-rich food such as cheese, sausages (Groenewald et al., 2014), oil-polluted soil (Wojtatowicz and Rymowicz, 1991) but also marine and hypersaline environments (Chi et al., 2009). Because this yeast produces large quantities of lipases it is able to utilize alkanes, fatty acids and also crude glycerol, the latter being the most convenient carbon source for *Y. lipolytica* in industrial applications. Because *Y. lipolytica* can naturally occurs in seawater, it has developed the molecular response pathway that mediates cellular adaptation to hyperosmotic stress. Under high osmotic stress *Y. lipolytica* produces erythritol, to protect the cells against harmful environmental conditions (Mirończuk et al., 2017; Rzechonek et al., 2017b). However, it was unknown if the growth of *Y. lipolytica* in seawater-based medium will be comparable with the growth in fresh water-based medium, especially strains industrially interested but isolated from other then marine and hypersaline environments. To this end, the wild-type strain A101 was grown in YPD medium and YPD-seawater medium, to verify its growth under two different conditions. As seen in Figure 1A, the growth of the strain under both conditions was not significantly different. Next, the same experiment was performed in medium containing glycerol as the main carbon source. Glycerol causes higher osmotic stress than glucose; thus the difference between fresh- and seawater might be significant. However, the growth of the strain A101 in seawater-based medium remained the same during 72 h (Figure 1B). This confirmed that *Y. lipolytica* could efficiently grow in rich medium based on seawater, even when glycerol was used as the main carbon source. Additionally, we check a growth of *Y. lipolytica* with sea salt concertation over average salinity of seawater (36 g/L) in world ocean. The growth of yeast was similar up to 40 g/L of sea salts in the medium, which correspond to highest salinity in world ocean. The longer lag phase was observed during the growth in medium containing 50 g/L of sea salts corresponding to brine water (Supplementary Figure S1).

However, *Y. lipolytica* accumulates high quantities of fatty acid under nitrogen starvation combined with excess of carbon (André et al., 2009). Therefore to verify whether seawater-based medium is suitable for SCO synthesis, a shake-flask experiment was performed.

### Evaluation of Lipid Accumulation by the Yeast *Y. lipolytica* in Seawater-Based Medium

As mentioned above, to induce accumulation of lipids in cells of *Y. lipolytica*, stress connected with depletion of the nitrogen source and excess of carbon in the environmental conditions are required. In the condition where a carbon source is available and nitrogen is absent the cells start to accumulate carbon in triglyceride (TGA) form in lipid bodies. Moreover, the content of TGAs in biomass of *Y. lipolytica* can be increased by metabolic engineering of the key enzymes in the Kennedy pathway (Abdel-Mawgoud et al., 2018). One of the crucial genes involved in SCO biosynthesis is the *DGA1* gene, encoding an acylCoA diacylglycerol acyltransferase (Qiao et al., 2015).

**FIGURE 2:**
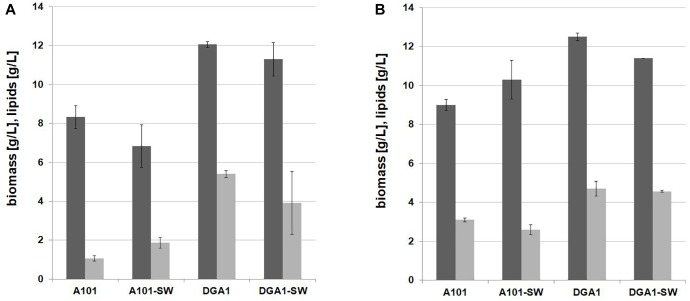
Shake-flask experiment for lipid (light gray bars) and biomass (dark gray bars) production by *Y. lipolytica* A 101 and AJD pAD-DGA1 strains grown in culture media containing glucose **(A)** and glycerol **(B)** as a carbon source prepared on freshwater or seawater basis (SW). The seawater based medium contain 36 g/L of sea salts mixture. Error bars indicate SD from three independent biological replicates.

First, the shake-flask experiment in seawater YNB medium supplemented with glucose was performed with the C/N ratio 100. In this experiment two different strains of *Y. lipolytica* were used: the strain AJD pAD-DGA1 overexpressing the *DGA1* gene was tested and as a control the wild-type A101 was used. In previous studies the TGA synthesis was performed in pH ranges from 5.0 to 6.0 (Papanikolaou et al., 2002, 2003; Dobrowolski et al., 2016), but this is also the optimal pH for CA synthesis (Sabra et al., 2017). Thus, to avoid undesired CA production pH of the medium was decreased to 3.0. As seen in Figure 2A, the wild-type and the engineered strain produced a similar titer of biomass in both media based on fresh- and seawater. The strain A101 produced 8.33 and 6.83 g/L of biomass in fresh- and seawater, respectively. For the strain AJD pADDGA1 the biomass titer was 12.07 and 11.3 g/L, for fresh- and seawater medium, respectively. In agreement with a previous study (Tai and Stephanopoulos, 2013; Kerkhoven et al., 2017), the strain overexpressing *DGA1* produced significantly more fatty acids (43.11 ± 3.6% of cellular dry weight [CDW]) than the wild-type (16.71 ± 1.38% of CDW). Moreover, seawater did not have a negative influence either on the growth or lipid biosynthesis by the engineered strain. The fatty acid profile was similar for both media, the major fatty acid that accumulated in the cells was oleic acid (C18:1), and it averaged around 60% for both strains. The strain overexpressing *DGA1* in seawater-based (SW) medium produced more (20%) palmitoleic acid (C16:1) than in fresh water-based (FW) medium (8%). Other fatty acids remained at a similar level (for details see Supplementary Table S1). Glycerol did not have a significant influence either on biomass or on SCO titer for both of the strains (Figure 2B). However, glycerol as a carbon source had an impact on the fatty acid profile. Again, the major fatty acid was oleic acid (C18:1), with amounts in the range 48–55%, although more stearic acid (C18:0) was produced, the level ranging from 22 to 25% of the total fatty acid pool. These results agree with other studies using *Y. lipolytica* grown on glycerol where oleic acid has been found in the highest concentration (Papanikolaou and Aggelis, 2002). This experiment showed that SW medium coupled to glycerol is a promising low-cost medium for fatty acid synthesis by *Y. lipolytica*. The engineered strain was able to grow and produce a high titer of fatty acid in medium based on glycerol and seawater. This effect was caused by changed genetic profile, *DGA1* overexpression results in higher lipid titer in the modified strain, in comparison to the wild-type. Thus the next step was investigation of crude glycerol application in this process.

### Production of SCO From Crude Glycerol in Seawater-Based Medium

**FIGURE 3:**
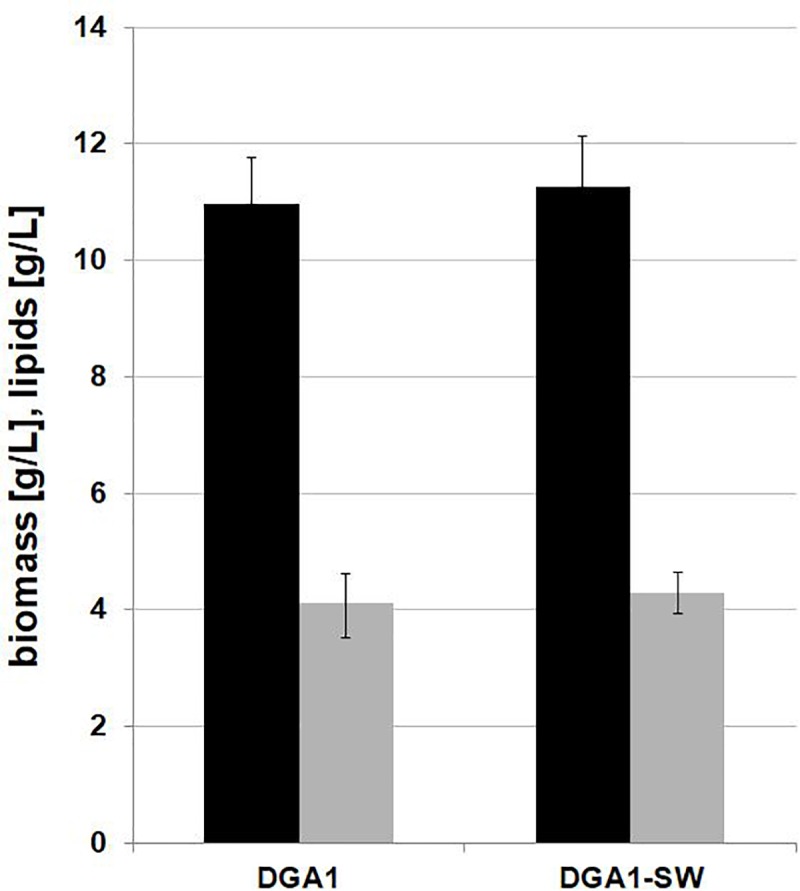
Shake-flask experiment for lipid (gray bars) and biomass (black bars) production by *Y. lipolytica* AJD pAD-DGA1 strain, grown in culture media containing crude glycerol prepared on freshwater or seawater basis (SW). The seawater based medium contain 36 g/L of sea salts mixture. Error bars indicate SD from three independent biological replicates.

Previous experiments have shown that the C/N ratio 100 in SW medium is appropriate for efficient lipid synthesis by *Y. lipolytica* A101, but for the strain AJD pADDGA1 with lipid overproduction, the C/N ratio can be lowered to 60, because of metabolism shifting to lipid biosynthesis without such high C/N stress, which is in line with other observations (Blazeck et al., 2014). The engineered strain did not reveal any delay in growth; on the contrary, it showed improved SCO synthesis, in view of which this strain was selected for the further experiments. It was previously demonstrated that despite many impurities such as methanol, salts, metals, and matter organic non-glycerol (MONG), crude glycerol is a good substrate for the yeast *Y. lipolytica* (Mirończuk et al., 2015; Dobrowolski et al., 2016; Rakicka et al., 2017). To decrease the cost of the SCO production, YNB medium was replaced with crude glycerol-based medium (FWCG, fresh water-crude-glycerol or SWCG, seawater-crude-glycerol medium) (for details see section “Materials and Methods”). The shake-flask experiment showed that crude glycerol coupled with seawater is a proper low-cost medium for fatty acid synthesis. A medium based on crude glycerol and fresh water was applied as a control (Figure 3). The yeast *Y. lipolytica* showed robust growth on the modified medium, and only a slightly lower biomass titer was obtained in comparison to YNB medium. Here, the strain AJD pADDGA1 obtained 11 g/L of biomass and the content of fatty acid was 37.38 ± 3.17% CDW for FW-CG medium and 38.04 ± 0.79% CDW for SW-CG medium. The seawater did not have any negative influence either on yeast growth or on lipid synthesis. Under both conditions, fresh and seawater-based medium, the *Y. lipolytica* strain produced the same level of biomass and SCO (Figure 3). The fatty acid level was 4 g/L for both media. In other studies conducted on crude glycerol, *Y. lipolytica* produced 5.5 or 6.1 g/L of lipids (Tchakouteu et al., 2015). However, it that study, crude glycerol with free-fatty acid was applied, what allowed for “ex novo” lipids synthesis. Regardless, this supports previous findings where the source of crude glycerol was identified as an essential factor of the lipid titer (Dobrowolski et al., 2016). In agreement with the previous experiment, the fatty acid profile remained unchanged. Oleic acid (C18:1) was the major fatty acid in the fatty acids pool (50%), followed by stearic acid (C18:0), which achieved 25%.

Given these results, we further tested the production of SCO in the bioreactor to scale up the process.

### Scale-Up Production of SCO From Seawater-Glycerol Based Medium in a Bioreactor

Previous results have shown that the newly established medium is appropriate for efficient fatty acid synthesis by the yeast *Y. lipolytica*. However, one of the main issues in biotechnology is scale-up of the process with maintenance of high parameters of fermentation such as titer, yield or productivity. Therefore, scale-up fermentation using crude glycerol as a carbon source and seawater with a 5 L fermenter was employed to better assess the potential for fatty acid production under selected conditions. The process took 96 h, and within this period the engineered strain was able to utilize 150 g/l of crude glycerol as a sole carbon source. As a control a fresh water-based and crude glycerol medium (FW-CG) was applied.

**FIGURE 4:**
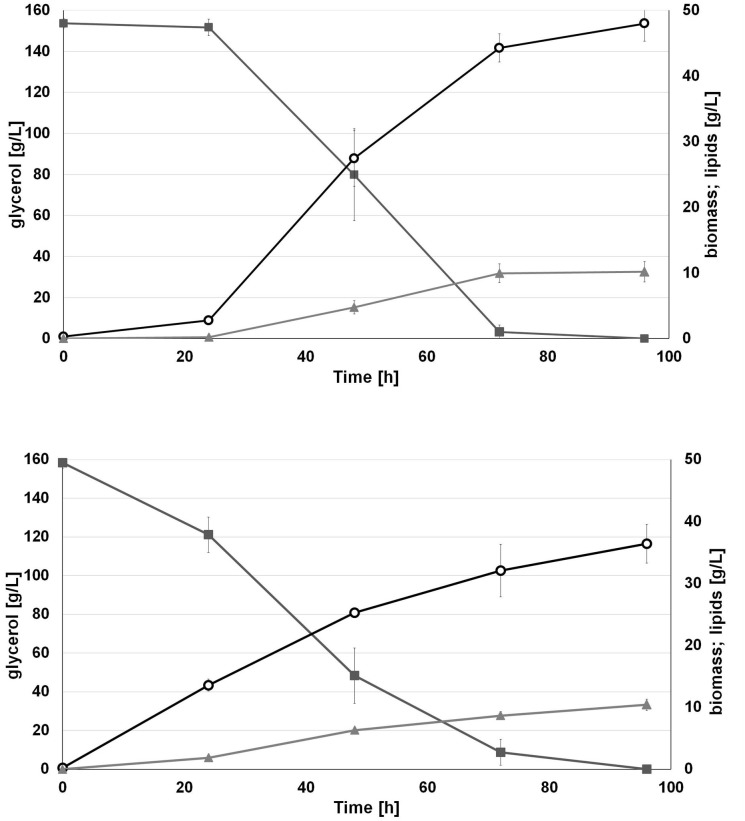
Batch bioreactor fermentation of *Y. lipolytica* AJD pAD-DGA1 strain in seawater (SW-CG upper panel) based medium and control-freshwater (FW-CG bottom panel) based medium. Time profiles of glycerol (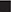), biomass (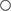), lipids (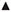). The seawater based medium contain 36 g/L of sea salts mixture. Error bars indicate SD from three independent biological replicates.

Interestingly, we observed that applied water has an influence on biomass production. As seen in Figure 4, the engineered strain in SW-CG medium showed an extended lag phase (Figure 4). Probably it was caused by higher osmotic stress induced by seawater and a high concentration of crude glycerol. However, in 48 h of growth, rapid growth was noted under these conditions. In agreement with previous experiments, the strain overexpressing *DGA1* did not show any significant delay in growth in SW-CG medium. The total amount of glycerol was utilized within 96 h, and the lipid titer was around 10 g/L for both media. Of note, in medium with seawater, the strain produced significantly more biomass than in fresh water medium. The most probably higher osmotic stress redirected carbon flux from glycerol to biomass instead of lipids. In freshwater medium, the carbon flux was equally distributed between biomass and lipid synthesis. The biomass titer at the end of the process was 36.4 and 48 g/L, for fresh- and seawater medium, respectively. The robust growth of the *DGA1* strain in SW-CG medium results in a lower percentage of lipids in biomass; it was 21.2 and 28.5% of CDW for SW-CG and FW-CG medium, respectively. These results are promising in comparison with other studies conducted on waste materials. The lipid percent in biomass obtained from food-derived volatile fatty acids was 18.23% (Gao et al., 2017). Moreover, when molasses was used as a carbon source in *Y. lipolytica* biomass only 14.75% of lipid was produced (Mirończuk et al., 2015). In other studies, where molasses and crude glycerol were applied in stepwise continuous fed-batch (SCFB) culture, the lipid content in biomass was 20% (100 g/L glycerol feeding) (Rakicka et al., 2015). In this study, during the bioreactor process the major fatty acid (around 50%) in the pool was oleic acid (C18:1). Next, the content of palmitic acid (C16:0), palmitoleic acids (C16:1) and stearic acid (C18:0) was in the range 11–13%, 12–9%, and 10–11%, respectively, for SW-CG and FW-CG (Table 1). Of note, the type of applied water did not have a significant influence on the fatty acid profile. Thus seawater is a good alternative to fresh water for medium preparation. Low pH of the medium significantly reduced the CA concentration in the medium, and at the end of the process the concentration of CA in the medium was 2.4 and 0.33 g/L for SW-CG and FW-CG medium, respectively.

**Table 1:** Fatty acid profile of strain AJD pAD-DGA1 in bioreactor studies.

Fatty acids	SW-CG medium	FW-CG medium
16:0	11.67 ± 0.28	13.18 ± 0.19
16:1	12.35 ± 1.05	9.07 ± 0.10
18:0	10.07 ± 0.67	11.20 ± 0.28
18:1	50.36 ± 1.10	49.5 ± 10.80
18:2	8.68 ± 0.21	10.96 ± 0.20
Total	93.13	93.93
Others	6.87	6.07
Y_X/S_ (g/g)	0.32	0.24
Y_L/X_ (g/g)	0.21	0.29
Y_L/S_ (g/g)	0.07	0.07


*SW-CG, seawater- crude-glycerol- based medium; FW-CG, freshwater- crude-glycerol- based medium; Y_*X/S*_, the biomass yield; Y_*L/X*_, the lipid yield from biomass; Y_*L/S*_, the lipid yield.*

This experiment proved that the medium based on seawater and crude glycerol is an appropriate, low-cost medium for fatty acid production by *Y. lipolytica*. The established medium is a good starting point for further optimization, including further metabolic engineering of the applied strain to improve and process optimization for enhancing SCO titer.

## Conclusion

Seawater is a suitable alternative to fresh water in media for SCO production via *Y. lipolytica*. Moreover, crude glycerol without any prior purification combined with seawater results in optimized low-cost medium. This medium allowed for high lipid accumulation in up to 21% of dry cell weight by the engineered strain. This is by far one of the highest lipid titers in *Y. lipolytica* biomass achieved via *de novo* synthesis from waste substrate and seawater. Application of seawater does not have a negative influence on the fatty acid profile. In summary, this study showed that *Y. lipolytica* in medium based on seawater could directly convert crude glycerol into SCO, which is beneficial for industrial application.

## Author Contributions

AD designed the study, constructed the overexpression cassette, performed bioreactor study, analyzed data and revised the manuscript. KD isolated fatty acids. PM performed fatty acids analysis. DR performed Bioscreen C experiments. AM wrote and revised the manuscript.

## Conflict of Interest Statement

The authors declare that the research was conducted in the absence of any commercial or financial relationships that could be construed as a potential conflict of interest.
