# Supplementary material for: Lipid Production From Waste Materials in Seawater-Based Medium by the Yeast Yarrowia lipolytica
Source: Front Microbiol. 2019 Mar 18;10:547. doi: 10.3389/fmicb.2019.00547 (PMC6431633; doi:10.3389/fmicb.2019.00547)
Supplement: Supplementary file 1 [file Data_Sheet_1.PDF]

Supplementary Material

Lipid production from waste materials in seawater-based medium by the yeast *Yarrowia lipolytica*

Adam Dobrowolski[1]*, Katarzyna Drzymała[1], Dorota A. Rzechonek[1], Paweł Mituła[2], Aleksandra M. Mirończuk[1]

*corresponding author

[1] Department of Biotechnology and Food Microbiology, Wroclaw University of Environmental and Life Sciences, Chełmońskiego 37, 51-630, Wrocław. Poland

[2] Institute of Environmental Engineering, Wroclaw University of Environmental and Life Sciences, Grunwaldzki sq 24, 50-363, Wrocław, Poland

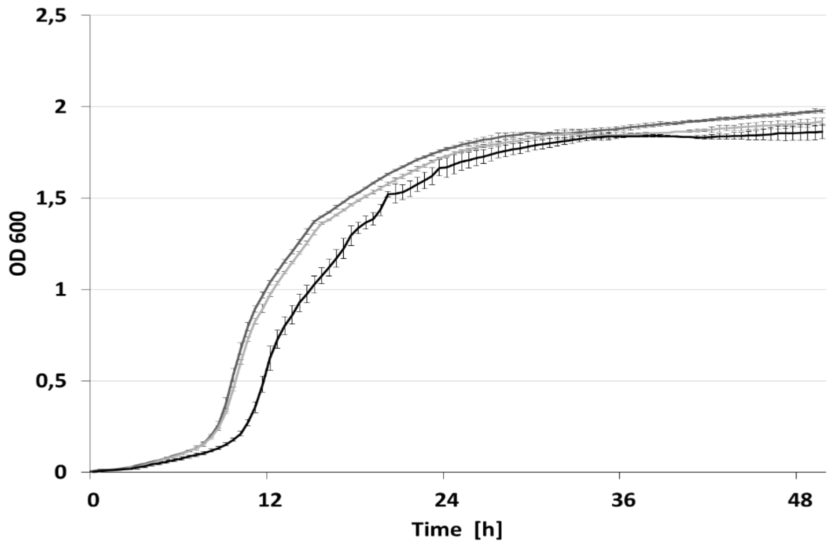

Supplementary Figure 1. Growth curves of *Y. lipolytica* A101 in medium based on seawater with salinity 36 g/L (dark gray), salinity 40 g/L (light gray) and 50 g/L (black). Strains were grown on YPD medium containing 2% glucose as carbon source. Experiment was performed at 28°C under constant agitation using Bioscreen C. The error bars present standard deviations.

Supplementary Table 1. Fatty acid profile (%) of lipid content produced by *Y. lipolytica* A101 and the engineered AJD pADDGA1 strains grown in culture media containing glucose or glycerol as a carbon source prepared on freshwater or seawater basis (SW) in shake-flasks experiment.

		16:0	16:1	18:0	18:1	18:2	others
GLUCOSE	**A101**	10.56	8.9	10.1	63.4	5.2	1.84
	**A101-SW**	12.45	8.27	11.95	59.85	5.72	1.76
	**DGA1**	9.63	8.28	16.12	59.5	4.51	1.96
	**DGA1-SW**	12.39	18.9	6.07	56.35	4.41	1.88
GLYCEROL	**A101**	16.83	4.08	24.3	49.02	3.67	2.1
	**A101-SW**	17.31	4.39	24.05	48.79	3.34	2.12
	**DGA1**	12.58	4.59	22.26	55	3.51	2.06
	**DGA1-SW**	15.15	3.93	25	50.59	2.92	2.41
